# Serum HMGB1 and Alcohol-Related Liver Disease

**DOI:** 10.3390/jcm15093397

**Published:** 2026-04-29

**Authors:** Iwona Popiolek, Piotr Hydzik, Krzysztof Ciszowski, Barbara Balicka-Slusarczyk, Ewa Gomolka, Beata Szkolnicka, Lubomir Skladany, Juan Pablo Arab, Ivica Grgurevic, Michal Kukla

**Affiliations:** 1 Department of Toxicology and Environmental Diseases, Jagiellonian University Medical College, Botaniczna 3, 31-503 Kraków, Poland; iwona.popiolek@uj.edu.pl (I.P.);; 2 Gastroenterology, Hepatology, Toxicology and Internal Medicine Clinical Department, University Hospital in Kraków, Jakubowskiego 2, 30-688 Kraków, Poland; 3 Toxicological Laboratory, Diagnostics Department, University Hospital in Kraków, Jakubowskiego 2, 30-688 Kraków, Poland; 4 Division Hepatology, Gastroenterology and Liver Transplantation, 2nd Department of Internal Medicine, Faculty of Medicine, Slovak Medical University, F. D. Roosevelt Teaching Hospital, 975 17 Banska Bystrica, Slovakia; 5 Departamento de Gastroenterología, Escuela De Medicina, Pontificia Universidad Católica De Chile, Santiago 8320165, Chile; 6 Division of Gastroenterology, Hepatology and Nutrition, Department of Medicine, Virginia Commonwealth University School of Medicine, Richmond, VA 23298, USA; 7 Department of Gastroenterology, Hepatology and Clinical Nutrition, University Hospital Dubrava, 10000 Zagreb, Croatia; 8 School of Medicine and Faculty of Pharmacy and Biochemistry, University of Zagreb, 10000 Zagreb, Croatia; 9 Department of Gastroenterology and Hepatology, Jagiellonian University Medical College, Jakubowskiego 2, 30-688 Kraków, Poland

**Keywords:** alcohol-related liver disease, alcohol hepatitis, HMGB1, alcohol use disorder

## Abstract

**Background/Objectives:** Alcohol-related liver disease (ALD) lacks widely adopted biomarkers that reflect disease activity and severity. High-mobility group box 1 (HMGB1), a damage-associated molecular pattern, has been implicated in ALD pathogenesis. We evaluated the detectability of circulating HMGB1 in patients with ALD during active alcohol use and examined clinical associations. **Methods:** In this observational study, we enrolled hospitalized adults with ongoing ethanol use between 1 November 2023, and 31 December 2024. Controls had no history of excessive alcohol consumption and normal liver biochemistry. Clinical features, laboratory tests, and severity scores (including MELD, MELD-Na, and CLIF-C AD) were recorded. Serum HMGB1 was measured by ELISA; values ≥ 0.08 ng/mL were considered detectable. **Results:** The cohort included 68 participants (58 with ALD and 10 controls); 29 patients had cirrhosis. HMGB1 was detectable in 32 measurements (42%), with a median concentration of 4.6 ng/mL (IQR, 0.78–10.6; range, 0.08–140.6). Detectable HMGB1 was more frequent in ALD than in controls (47% vs. 11%). Compared with HMGB1-negative patients, HMGB1-positive patients had higher total bilirubin and creatinine levels, prolonged activated partial thromboplastin time, higher white cell counts, and lower serum sodium. Liver enzyme activities and INR did not differ meaningfully by HMGB1 status. MELD, MELD-Na, and CLIF-C AD scores were higher in HMGB1-positive patients. Admission ethanol levels were higher in HMGB1-negative patients. Mortality and readmission did not differ by HMGB1 status. **Conclusions:** Detectable circulating HMGB1 is present in a subset of patients with ALD and is associated with greater liver disease severity.

## 1. Introduction

Alcohol-related liver disease (ALD) refers to a spectrum of hepatic conditions caused by excessive alcohol consumption, ranging from isolated hepatic steatosis through severe alcohol-related hepatitis (AH) and cirrhosis [1]. ALD exhibits a dose-dependent relationship: the higher the lifetime alcohol intake the greater the likelihood of developing hepatic steatosis, which may progress to AH, fibrosis, cirrhosis, liver failure and death [2]. Cirrhosis is also a risk factor for hepatocellular carcinoma [3]. Furthermore, ALD has emerged as one of the leading causes of advanced liver disease globally, and alcohol-related cirrhosis is now a major indication for liver transplantation [4]. A meta-analysis [5] showed that even a one drink/day consumption increased the risk of cirrhosis in women, whereas intakes of 5–6 drinks/day significantly heightened the risk both in women and men [1,6].

The pathophysiological mechanisms underlying ALD are complex, involving both direct cellular toxicity of ethanol and the resultant inflammatory and fibrotic responses. Ethanol intake is cause of factors such as oxidative stress, inflammation, and apoptosis [7,8,9,10]. One of the mechanisms involves the activation of hepatocytes by alcohol metabolites, lipids, and damage-associated molecular patterns (DAMPs) [11]. One of these DAMPs is high-mobility group box 1 (HMGB1). HMGB1 is a highly conserved nuclear protein involved in gene expression regulation, DNA repair, and cellular stress responses [12,13,14,15]. In addition to its nuclear functions, ubiquitous HMGB1 has the ability to act as a pro-inflammatory cytokine upon extracellular release, particularly in the context of tissue injury [16]. Under stress conditions such as trauma, ischemia, infection or excessive alcohol intake. HMGB1 is secreted by activated macrophages and other cells of the immune system and acts as DAMP. HMGB1 may also undergo post-translational modifications (PTMs) in response to alcohol consumption. It plays a pivotal role in alcohol-related liver disease (ALD) due to its involvement in inflammatory and immune responses [17]. Another key regulator is Sirtuin 1 (SIRT1), an NAD^+^-dependent deacetylase known for its roles in cellular stress responses and metabolic regulation. SIRT1 modulates HMGB1 acetylation and translocation, thereby attenuating alcohol-induced liver injury (ALI) [18]. Extracellular HMGB1 can interact with various receptors, such as Toll-like receptors (TLR) and the receptor for advanced glycation end products (RAGE), leading to the activation of pro-inflammatory pathways [19,20,21]. The release of HMGB1 is often associated with increased cytokine production, including tumor necrosis factor-alpha (TNF-α), interleukin-1β (IL-1 β) and interleukin-6 (IL-6), amplifying the inflammatory response [22,23].

Previous studies suggest that HMGB1 functions as a key mediator in both chronic and acute liver diseases, including metabolic dysfunction-associated steatotic liver disease (MASLD), hepatocellular carcinoma (HCC), and ischemia–reperfusion injury [24]. Increased secretion of HMGB1, especially in central nervous system has also been reported in individuals consuming ethanol [25,26,27]. In addition, Ye et al. explored the mechanisms through which HMGB1 is released from hepatocytes under pathological conditions, implicating oxidative stress giving its contribution to processes such as pyroptosis and inflammation in alcohol-associated liver pathology [28]. Liver biopsies from patients with ALD demonstrated a marked increase in HMGB1 expression and translocation, which correlated with disease stage, compared to healthy controls [29]. Activation of HMGB1 and subsequent downstream signaling pathways are contributing factors in the pathogenesis of MASLD, ALD, and drug-induced liver injury (DILI) [19,30]. HMGB1 expression is increased and undergoes PTMs in response to alcohol consumption [29]. Experimental evidence indicates that oxidized HMGB1 of hepatocyte origin can act as a ligand for TLR4 signaling in myeloid cells, promoting steatosis, inflammatory cell infiltration, and IL-1β production in ALD. In contrast, acetylated HMGB1 may offset the deleterious effects of oxidized HMGB1 in alcohol-associated liver disease [31]. Moreover, elevated HMGB1 levels have been reported in the presence of active inflammatory processes [32,33] and acute-on-chronic liver failure [34] and were associated with greater ALD severity [35]. In summary, HMGB1 is an alarmin implicated in the pathogenesis of multiple liver diseases. Its use as a biomarker for liver disease in those with alcohol use disorder (AUD) has not been studied. The predictive potential of HMGB1 was validated in an independent cohort of patients with AUD [35]. Serum HMGB1 shows promise as a biologically relevant biomarker for ALD in patients with AUD.

Given the important role of HMGB1 in alcohol-related liver injury, this molecule has been proposed as a potential therapeutic target in ALD [1,36]. Indeed, advanced ALD—particularly severe AH—has a poor prognosis and limited treatment options, highlighting the need for novel therapies (for example, approaches to stimulate liver regeneration) [37]. In this study, we aimed to investigate the associations between serum HMGB1 levels and clinical features of ALD in patients with recent alcohol intake.

## 2. Materials and Methods

The study included adult patients with alcohol use disorder (AUD), defined as chronic excessive alcohol consumption (mean daily ethanol intake > 40 g for women or >50 g for men), with or without evidence of alcohol-related liver disease (ALD), defined by elevated liver enzymes or characteristic imaging findings (ranging from steatosis to cirrhosis). The control group consisted of patients without a history of excessive alcohol use and with normal liver function, who were hospitalized for routine screening endoscopic examinations.

Exclusion Criteria included were: age < 18 years, pregnancy or lactation, DILI, ischemic hepatitis, biliary obstruction, active viral hepatitis, autoimmune hepatitis, or Wilson’s disease, HCC outside the Milan criteria, extrahepatic malignancies with an expected survival of less than 6 months, history of severe extrahepatic disease (e.g., chronic renal failure requiring hemodialysis, severe heart disease classified as NYHA class ≥ 3, or advanced chronic pulmonary disease with expected survival under 6 months), and human immunodeficiency virus (HIV) infection.

Patients were divided into three subgroups, according to [35]:

- Non-severe ALD: patients admitted for alcohol detoxification and/or treatment of withdrawal symptoms with a diagnosis of AUD, abnormalities in liver parameters and without evidence for cirrhosis based on clinical documentation, laboratory values, hepatic imaging, or biopsy with MELD < 18;
- Severe ALD: patients with a diagnosis of AUD and either severe AH (MELD ≥ 18) or evidence for cirrhosis based on clinical documentation, hepatic imaging, or biopsy;
- Control group: patients without evidence of liver injury in laboratory or imaging studies, hospitalized for routine diagnostic procedures (screening gastrointestinal endoscopy).

Diagnosis of AH was based on clinical (recent onset of jaundice) and typical laboratory findings (AST > 50 IU/L) in a patient with a history of heavy alcohol use [1].

Upon admission, patients underwent a comprehensive evaluation, including physical examination and a structured interview, to collect data on age, sex, body mass index (BMI), comorbidities, medications, tobacco use and history of alcohol consumption.

Esophageal varices were confirmed by endoscopy. Hepatic encephalopathy was described according to the West Haven grading system upon admission. Ascites was confirmed by ultrasonography during hospitalization. Ethanol concentrations were determined either in blood, using a routine enzymatic assay (EMIT II Plus Ethyl Alcohol Assay), or in exhaled air, using a calibrated detection device.

### 2.1. Blood Samples Collection and Analyses

Venous blood samples were collected during hospitalization. Samples were sent to the Certified Diagnostics Unit of the University Hospital for analysis, which included: complete blood count; basic renal and liver function parameters (albumin, bilirubin, creatinine concentrations; alkaline phosphatase [ALP], alanine aminotransferase [ALT], aspartate aminotransferase [AST], gamma-glutamyl transferase [GGT] activity); coagulation parameters (prothrombin time [PT], international normalized ratio [INR], activated partial thromboplastin time [APTT]); inflammatory markers (C-reactive protein [CRP], procalcitonin, lactate concentrations); electrolytes.

Liver disease severity scores were calculated as follows: Model for End-Stage Liver Disease (MELD) [38], MELD-Na [39], MELD 3.0 [40,41], The Age-Bilirubin-INR-Creatinine Score (ABIC) [42], NAFLD-Score [43], Fibrosis-4 (FIB-4) index [44], The CLIF Consortium Organ Failure (CLIF-C OF) Score, The CLIF Consortium Acute Decompensation (CLIF-C AD) Score, The CLIF Consortium Acute-On-Chronic Liver Failure (CLIF C ACLF) [45,46,47], Child–Pugh Score [48,49], Maddrey’s modified discriminant function (mDF) [50].

Plasma concentration of HMGB1 was measured using human HMGB1 Sandwich ELISA kit (cat. no. KE00343, Proteintech, Rosemont, IL, USA) following the manufacturer’s instructions. Samples were processed according to standardized procedures by fully qualified laboratory diagnosticians. Plasma samples (30 µL) were diluted 1:1 or 1:5 and measured colorimetrically at a wavelength of 450 nm. Concentrations were expressed in ng/mL with a sensitivity of 0.04 ng/mL (data provided by the manufacturer). Cutoff concentration was set to 0.08 ng/mL, because of the lowest dilution. Results below this value were considered negative. Outlier measurements were repeated. The intra-assay and inter-assay coefficients of variation (CVs) were below 9.9% and 9.5%, respectively.

### 2.2. Statistical Methods

Results were presented as numbers (percentages, %) for categorical variables or as medians with interquartile ranges (IQR) for continuous variables. Normality of data distribution was tested using the Kolmogorov–Smirnov test. Assignment to particular groups was considered as an ordinal variable. Quantitative variables were compared using the Mann–Whitney U test. Associations between categorical variables were analyzed with the χ^2^ test. Categorical variables were presented as numbers (percentages, %) and compared using the χ^2^ test or Fisher’s exact test, as appropriate. Spearman’s rank-order correlation coefficient was used to determine relationships between biomarkers.

The significance level (α) was set at 0.05. Statistical analyses were conducted using Statistica software (version 13, TIBCO Software Inc., Tulsa, OK, USA).

## 3. Results

The study cohort consisted of 68 adult patients admitted to internal medicine department between 1 November 2023, and 31 December 2024. The median age was 46 years (range: 22–74 years). Five patients required more than one hospitalization during the study period. Comorbidities included type 2 diabetes mellitus diagnosed in eight patients and arterial hypertension in 15 patients. A total of five deaths were recorded during observation period. Current or former tobacco use was documented in 48 patients. In study group, 29 patients had liver cirrhosis, 22 patients were diagnosed with alcohol hepatitis (AH), 17 of them had cirrhosis and AH.

HMGB1 was detectable in 32 serum samples (42% of all measurements, including repeat admissions). Among HMGB1-positive samples, concentrations ranged from 0.08 to 140.6 ng/mL, with a median of 4.6 ng/mL (IQR 0.78–10.6 ng/mL).

Table 1 summarizes the baseline characteristics of the ALD patients (*n* = 58) and controls (*n* = 10). A positive HMGB1 test was significantly more frequent in ALD patients than in controls (47% vs. 10%, *p* < 0.05). Age, BMI, and the prevalence of smoking, diabetes, and hypertension did not differ significantly between the two groups. However, the ALD group had a much higher proportion of males (67% vs. 20% in controls, *p* < 0.01).

As expected, the ALD patients demonstrated laboratory abnormalities consistent with chronic liver injury. They had significantly lower platelet counts, hemoglobin levels, and serum sodium (and other electrolyte) concentrations compared to controls. Liver enzyme levels (ALT, AST, GGT, ALP) were markedly higher in the ALD group than in controls (Table 1). Creatinine and WBC were the only parameters that did not differ significantly between the ALD and control groups. Not surprisingly, the extent of alcohol exposure was far greater in the ALD patients; for example, the median duration of the current drinking episode was 13.5 weeks (IQR 3–104 weeks) in the ALD group, versus zero in controls.

### 3.1. HMGB1 and Basic Parameters

The study cohort was stratified into two groups based on detectable versus non-detectable HMGB1 concentrations (HMGB1-negative (−), *n* = 35; HMGB1-positive (+), *n* = 31). Baseline demographic, clinical, and laboratory parameters are summarized in Table 2.

No significant differences were observed between these two groups in age, sex distribution, BMI, infection or smoking status. Similarly, rates of death and hospital readmission did not differ significantly between groups (Table 2).

Vital signs at admission (heart rate, systolic and diastolic blood pressure) were also similar between HMGB1-negative and HMGB1-positive patients.

With respect to laboratory findings, HMGB1-positive patients had slightly but significantly higher serum creatinine levels and lower sodium levels than HMGB1-negative patients. They also exhibited a prolonged APTT, whereas platelet counts and INR did not significantly differ between the groups (Table 3). Other hematologic indices (e.g., neutrophil and lymphocyte counts, hemoglobin) and inflammatory markers (CRP and procalcitonin) showed no significant inter-group differences.

### 3.2. HMGB1 and Liver Condition

There were no significant differences between HMGB1-positive and -negative patients in standard liver biochemistry: ALT, AST, GGT, and ALP activities and albumin concentrations were similar in the two groups (Table 3). The only basic laboratory parameter that differed was total bilirubin, which was significantly higher in the HMGB1-positive group.

Composite liver disease severity scores, however, demonstrated notable divergence. The median MELD (16.5 vs. 9.4, *p* < 0.05) and MELD-Na (18.8 vs. 10.9, *p* < 0.05) scores were both significantly higher in HMGB1-positive patients than in HMGB1-negative patients, reflecting more advanced hepatic dysfunction. Likewise, the HMGB1-positive group had a higher median CLIF-C AD score (*p* < 0.05). By contrast, other prognostic indices—including MELD 3.0, ABIC, FIB-4, NAFLD fibrosis score, CLIF-C ACLF, and Child–Pugh class—did not differ significantly between the two groups.

Median FIB-4 reflected high risk of advanced fibrosis (>2.67) [44] without significant difference in both groups. Notably, when applying an ABIC cutoff of 6.71, a significantly greater proportion of HMGB1-positive patients fell into the intermediate- or high-risk category for 90-day mortality [42].

Analysis of clinical features revealed similar trends. The prevalence of cirrhosis was higher in the HMGB1-positive group (58% vs. 31%), and HMGB1-positive patients experienced hepatic encephalopathy and esophageal varices more frequently than HMGB1-negative patients. However, these differences did not reach statistical significance. Rates of jaundice and ascites were comparable between the groups. Only eight out of 66 patients (12%) developed acute-on-chronic liver failure (ACLF) during hospitalization, with no difference in ACLF occurrence between HMGB1-positive and -negative patients

### 3.3. HMGB1 and Alcohol Consumption

Alcohol use patterns differed between the groups. At admission, blood ethanol levels were significantly higher in HMGB1-negative patients (Table 4), and an admission blood alcohol test was positive in a greater fraction of HMGB1-negative patients. Additionally, HMGB1 concentration was significantly negatively correlated with ethanol levels (R = −0.36, *p* < 0.05). Conversely, the HMGB1-positive patients had a longer abstinence period prior to admission (by study design, all controls had no recent alcohol use.) Other drinking-related parameters—the duration of the drinking episode leading up to admission, average daily alcohol intake, incidence of alcohol withdrawal syndrome, and frequency of high-intensity drinking—did not differ significantly between HMGB1-positive and -negative patients.

Importantly, we observed that admission blood ethanol concentration was inversely correlated with markers of inflammation and liver injury across the cohort. Ethanol levels were negatively correlated with WBC (R = −0.36, *p* < 0.01), monocyte count (R = −0.41, *p* < 0.05), CRP (R = −0.54, *p* < 0.001), total bilirubin (R = −0.55, *p* < 0.001), and INR (R = −0.47, *p* < 0.001). Conversely, the duration of pre-admission abstinence showed significant positive correlations with these and other indicators of inflammation or organ dysfunction: abstinence days correlated directly with WBC (R = 0.27, *p* < 0.05), monocyte count (R = 0.35, *p* < 0.05), APTT (R = 0.54, *p* < 0.0001), INR (R = 0.61, *p* < 0.0001), CRP (R = 0.49, *p* < 0.0001), total bilirubin (R = 0.53, *p* < 0.0001), serum glucose (R = 0.30, *p* < 0.05), and creatinine (R = 0.27, *p* < 0.05). In other words, patients who had been abstinent for at least several days before admission tended to have higher inflammatory markers and more significant liver/kidney dysfunction, whereas those who presented acutely intoxicated had suppressed inflammatory profiles. Notably, ALT activity was the only parameter negatively correlated with the length of abstinence (R = −0.34, *p* < 0.01), consistent with liver enzyme levels decreasing during short-term sobriety.

## 4. Discussion

In this observational cohort of hospitalized adults with ongoing alcohol use, we found that circulating HMGB1 was detectable in a substantial proportion of patients with alcohol-related liver disease and was associated with markers of more advanced disease. Consistent with our results, elevated HMGB1 levels have been reported in patients with ALD [35,51,52]. Specifically, HMGB1 detectability was linked to higher bilirubin and creatinine, prolonged APTT, higher WBC count, lower sodium, and higher prognostic scores (MELD and MELD-Na and CLIF-C AD), as well as a higher prevalence of cirrhosis and portal hypertension complications (esophageal varices and encephalopathy). Mortality and readmission did not differ by HMGB1 status in this sample.

Importantly, our findings should be interpreted in the context of the biological characteristics of HMGB1. This molecule represents a damage-associated molecular pattern released during systemic inflammation, tissue injury, and immune activation. Therefore, the observed association between HMGB1 detectability and higher disease severity likely reflects overall disease burden and systemic inflammatory activity rather than liver-specific injury alone. Extracellular HMGB1 is an alarmin derived either by active secretion by innate immune cells (e.g., monocytes), or by passive release by dead, dying or injured cells such as hepatocytes and cancer cells [53].

On the other hand, HMGB1 is highly induced during liver injury. Recent studies have identified specific post-translational modifications that may influence its biological effects in the liver, and hepatocytes appear to be a major source of these HMGB1 isoforms. Additionally, Ge et al. demonstrated a progressive increase in HMGB1 expression with advancing disease stage in liver biopsies and serum from patients with alcohol-related steatohepatitis [52]. This phenomenon has also been confirmed in experimental murine models of liver fibrosis [52,54,55].

Patients with detectable HMGB1 levels had a higher mortality risk as assessed by the CLIF-C AD and ABICs. In the HMGB1-negative group, the median CLIF-C AD score corresponded to a low risk of death (3-month mortality < 2%), whereas in HMGB1-positive patients, the median score reflected an intermediate mortality risk (3-month mortality 2–30%) [47]. The median Child–Pugh score in both groups corresponded to Child–Pugh class B, indicating significant hepatic functional impairment. Only a minority of patients admitted to the hospital fulfilled criteria for acute-on-chronic liver failure (ACLF), which may partly explain the low prevalence of detectable HMGB1 concentrations [35,53].

However, a recent study in ALD revealed that mortality prediction models can significantly differ from real-life mortality [56]. The higher prevalence of cirrhosis and elevated CLIF-C AD scores in patients with detectable HMGB1 levels are consistent with the increased frequency of esophageal varices and hepatic encephalopathy. HMGB1 is released during inflammation and can readily cross the blood–brain barrier, where it has been implicated in various forms of encephalopathy [57,58], including hepatic encephalopathy [59]. In a study comparing HMGB1 concentrations in peripheral and portal venous blood in patients undergoing TIPS procedures, higher HMGB1 levels were observed in patients with portal hypertension. These findings were attributed to well-developed collateral circulation and increased influx of HMGB1 originating from congested intestines and the spleen [59]. This mechanism may explain the higher prevalence of detectable HMGB1 levels in patients with esophageal varices observed in the present study.

Vilela et al. evaluated positive HMGB1 levels in cirrhotic patients with variceal bleeding [60] and subsequent studies assessed the performance of HMGB1 as a predictor of outcomes following variceal bleeding in patients with advanced chronic liver disease [61]. Regarding renal function, a positive association between HMGB1 levels and the prevalence of acute kidney injury in ICU patients and individuals with decompensated liver cirrhosis has been described previously [60,61,62]. Coagulation abnormalities in relation to HMGB1 have also been investigated; positive correlations between damage-associated molecular patterns (DAMPs), including HMGB1, and disseminated intravascular coagulation have been reported in critically ill patients [63,64], but there are reports not confirming significant association with prolonged APTT [65,66].

HMGB1-positive patients exhibited features of liver damage with multisystem dysfunction, including renal impairment and electrolyte disturbances, which are incorporated into composite prognostic scores.

Consistent with previous studies, we did not observe significant differences in standard liver enzyme activities (ALT, AST, GGT) between HMGB1-positive and HMGB1-negative patients. Extracellular HMGB1 is derived either by active secretion by innate immune cells (e.g., monocytes), or by passive release by dead, dying or injured such as hepatocytes and cancer cells [53].

A notable observation in our study is the relationship between alcohol exposure and HMGB1 detectability. Patients who were actively intoxicated at admission had lower HMGB1 levels, whereas those with longer periods of abstinence were more likely to have detectable HMGB1. Moreover, ethanol levels were inversely correlated with inflammatory markers, including monocyte count (monocytes are a key source of HMGB1) [67]. These findings are consistent with experimental and clinical data suggesting that acute alcohol exposure suppresses immune responses [68,69,70,71,72], while inflammatory activation may increase during early abstinence [70,73,74,75]. This highlights the importance of timing when interpreting HMGB1 measurements in patients with alcohol use disorder and suggests that biomarker assessment may be more informative after a period of abstinence. Despite numerous reports describing release of HMGB1 after chronic drinking, experimental studies in binge-drinking rat models revealed that significant increases in circulating HMGB1 concentrations can be observed after seven days of abstinence rather than immediately following alcohol exposure [76].

Several methodological aspects of our study should be considered. First, HMGB1 was measured as total circulating protein using a single time-point assessment. Longitudinal assessment of HMGB1 would add some information about temporal relationships and dynamic changes during disease progression or recovery. We did not differentiate between redox isoforms of HMGB1 as well as PTMs, which are known to have distinct biological functions in ALD [29], nor did we assess temporal changes during hospitalization. In future studies should evaluate specific HMGB1 isoforms to better understand their biological and clinical relevance.

Second, although inflammatory markers such as CRP and procalcitonin were measured and infection status was analyzed in our cohort, we did not assess concentrations of key cytokines (TNF-α, IL-1β, and IL-6), which may influence or correlate with HMGB1 levels.

Third, the study population was heterogeneous, encompassing a broad spectrum of alcohol-related liver disease, from non-severe forms to advanced cirrhosis. The diagnosis of liver disease was established on the basis of clinical symptoms, laboratory findings, and radiological confirmation, but without histopathological verification. Information on alcohol consumption patterns, including duration and average daily intake, was obtained from patient interviews and psychological consultations rather than standardized questionnaires. As the vast majority of patients had consumed excessive amounts of alcohol for many years, they were often unable to provide precise estimates of daily intake due to memory impairment or alcohol-related palimpsests. There could be a bias related to the fact that patients with diagnosed and symptomatic chronic liver failure consume less alcohol and have longer abstinence periods because of their general condition. Unfortunately, the awareness of liver disease is still the main motivator for abstinence [77].

While this heterogeneity may limit mechanistic interpretation, it reflects real-world clinical practice in hospitalized patients with alcohol-related liver disease.

Fourth, the control group was relatively small and characterized by an inverted male-to-female ratio. Therefore, comparisons with controls should be interpreted with caution. However, previous studies have been successfully conducted using similarly sized populations [51].

Nevertheless, despite these limitations, our study provides new insights into HMGB1’s role in patients with alcohol-associated liver disease.

In addition, our analytical approach was based on dichotomization of HMGB1 detectability, which reflects the lower limit of assay sensitivity. Biological variability of population may contribute to the wide range of HMGB1 concentrations. While this strategy allowed robust group comparisons, it may have reduced statistical power and limited the ability to explore dose–response relationships.

Despite these limitations, our study provides clinically relevant insights. HMGB1 detectability was consistently associated with established prognostic scores and markers of disease severity, suggesting that it may reflect clinically meaningful processes in patients with alcohol-related liver disease. However, given its lack of specificity and the absence of analysis of specific isoforms, HMGB1 should not be interpreted as a standalone biomarker of liver injury, but rather as a potential indicator of systemic disease severity and inflammatory burden.

## 5. Conclusions

In conclusion, circulating HMGB1 is detectable in a subset of patients with alcohol-related liver disease and is associated with markers of more advanced disease and higher prognostic scores. These findings suggest that HMGB1 reflects overall disease severity and systemic inflammatory activity rather than liver-specific injury.

Our results also indicate that the detectability of HMGB1 is influenced by recent alcohol exposure, with lower levels observed during acute intoxication and higher levels after a period of abstinence. This highlights the importance of timing when interpreting HMGB1 measurements in this population.

Given its biological characteristics and the limitations of the present study, HMGB1 should be considered a non-specific marker of systemic inflammation and disease severity rather than a diagnostic biomarker for alcohol-related liver disease. Further studies are needed to evaluate its clinical utility, including longitudinal assessments, analysis of specific HMGB1 isoforms, and adjustment for infectious and inflammatory confounders.

## Figures and Tables

**Table 1:** Basic comparison for study and control group. Where applicable, *p* < 0.05 from U-Mann–Whitney test or Chi-square test were considered significant. IQR—interquartile range. The results are shown as medians (IQR) or counts and percentages.

Variables	Study Group (*n* = 58)	Control (*n* = 10)	*p*-Value
Age, years	46 (39–53)	46.5 (42–51)	0.83
Body mass index, kg/m^2^	24.2 (21.3–27.1)	25.7 (22.4–29.0)	0.36
Male	39 (67%)	2 (20%)	*p* < 0.01
Current smoking n (%)	37 (65%)	5 (50%)	0.37
Diabetes, n (%)	6 (10%)	2 (20%)	0.39
Hypertension, n (%)	13 (22%)	2 (20%)	0.87
WBC, 10^3^/μL	6.9 (5.4–12)	6.7 (4.8–7.1)	0.25
Hemoglobin, g/dL	12.6 (10.4–14.3)	13.5 (13.2–14.8)	*p* < 0.05
MCV, ft	93 (87–98)	89 (85–90)	*p* < 0.05
PLT, 10^3^/μL	117.5 (85–173)	267 (221–293)	<0.001
Creatinine, μmol/L	64.1 (56.2–82.8)	67.9 (63.2–74.1)	0.65
Sodium, mmol/L	137 (133–139)	140 (139–140)	*p* < 0.01
Potassium, mmol/L	3.9 (3.5–4.3)	4.3 (4.3–4.4)	*p* < 0.01
CRP, mg/L	10.9 (4.6–39.2)	3 (0.6–4.8)	*p* < 0.01
ALT, U/L	50 (31–108)	22 (14–33)	*p* < 0.01
AST, U/L	124 (68–195)	25 (21–33)	<0.0001
GGT, U/L	251 (128–875)	16 (11–23)	<0.001
ALP, U/L	148 (101–238)	75 (64–85)	*p* < 0.05
Albumin, g/L	31 (27–40.5)	43 (40–46)	*p* < 0.05
Bilirubin, μmol/L	30 (11–95)	9 (7–17)	*p* < 0.01
INR	1.2 (1–1.6)	1 (1–1.1)	*p* < 0.01
APTT, s	33 (28–40)	27 (26–29)	*p* < 0.01
Week alcohol intake, grams	1100 (540–1630)	0 (0–20)	<0.0001
Positive test for HMGB1	27 (47%)	1 (11%)	*p* < 0.05

**Table 2:** Basic characteristics of the patients with negative or positive HMGB1 concentration. Where applicable, *p* < 0.05 from U-Mann–Whitney test or Chi-square test were considered significant. IQR—interquartile range. The results are shown as medians (IQR) or counts and percentages.

Variable	HMGB1 Negative *n* = 35	HMGB1 Positive *n* = 31	*p*-Value
Age, years	46 (36–52)	45 (38–53)	0.80
Male n (%)	21 (60%)	23 (74%)	0.22
Death n (%)	2, 6%	3, 10%	0.54
Readmission n (%)	10, 29%	4, 13%	0.12
Smoking n (%)	20, 57%	21, 68%	0.46
Infection n (%)	8, 23%	6, 19%	0.73
BMI, kg/m^2^	24 (21.3–27.5)	24.2 (21–27.1)	0.92
SBP, mmHg,	138.5 (122–149)	132 (114.5–146.5)	0.79
DBP, mmHg,	84 (73–94)	86 (72–93.5)	0.60
HR, bpm,	92.5 (79–104)	95.5 (73–105)	0.51
WBC, 10^3^/μL	6.3 (5.2–8)	10.5 (5.4–13.3)	*p* < 0.05
Hemoglobin, g/dL	12.7 (10.9–13.8)	12.5 (9–14.3)	0.55
Lymphocytes, 10^3^/μL	1.1 (1–2.1)	1.3 (1.2–2)	0.56
Neutrophiles, 10^3^/μL	4.1 (3.4–8.4)	8 (3.2–10.7)	0.33
Monocytes, 103/μL	0.6 (0.5–0.8)	0.8 (0.5–1)	0.12
PLT, 10^3^/μL	109 (67–173)	126 (99–177)	0.60
CRP, mg/L	10.5 (4–34)	11.3 (4.7–42.8)	0.25
Procalcitonin, ng/mL	0.4 (0.1–1.1)	0.3 (0.2–1.4)	0.30
Creatinine, μmol/L	61 (54–77)	73 (59–90.5)	*p* < 0.05
Sodium, mmol/L	137 (135–140)	136 (133–138)	*p* < 0.05
Potassium, mmol/L	3.9 (3.4–4.4)	3.9 (3.6–4.3)	0.53
Glucose, mmol/L	5 (4.7–6.3)	5.7 (5–6.6)	0.18
APTT, s	30 (27–38)	36 (31–43)	*p* < 0.05

Abbreviations: BMI, body mass index; bpm, beats per minute; CRP, CRP-reactive protein; DBP, diastolic blood pressure; HR, heart rate; PLT, platelets; SBP, systolic blood pressure; WBC, white blood cells.

**Table 3:** Characteristics of patients’ liver condition with negative or positive HMGB1 concentration. Where applicable, *p* < 0.05 from U-Mann–Whitney test or Chi-square test were considered significant. IQR—interquartile range. The results are shown as medians (IQR) or counts and percentages.

Variable	HMGB1 Negative *n* = 35	HMGB1 Positive *n* = 31	*p*-Value
ALT, U/L	63 (31–114)	46 (30–99)	0.66
AST, U/L	142 (72–245)	106 (67–158)	0.91
GGT, U/L	287 (140–1503)	220 (88–586)	0.92
ALP, U/L	128 (100–326)	159 (106–214)	0.42
Albumin, g/L	31 (28–40)	31 (27–42)	0.37
Bilirubin, μmol/L	25 (9–49)	36 (13–194)	*p* < 0.05
INR	1.1 (1–1.6)	1.4 (1–1.7)	0.07
MELD	9 (7–13)	16.5 (9–22)	*p* < 0.05
MELD-Na	11 (7.5–16)	19 (9–24)	*p* < 0.05
MELD-3.0	15 (9–20)	19 (11–25)	0.14
ABIC	6.1 (5.2–6.7)	6.9 (5.7–7.5)	0.11
ABIC > 6.71	9, 26%	16, 52%	*p* < 0.05
Fib-4	8.4 (3.2–17.4)	4.9 (2.4–9.4)	0.9
NAFLD-Score	1.9 (0.6–2.9)	0.9 (−0.5–2)	0.25
CLIF Organ Failure Score	6 (6–7)	7 (6–8)	0.06
CLIF-C AD Score	39 (35.5–50.5)	49 (41–58)	*p* < 0.01
CLIF-C ACLF	30 (27–37)	33.5 (29–43)	0.08
ACLF 1–3 occurence	3, 9%	5, 16%	0.35
Child–Pugh Score	8 (5–9)	9 (5–11)	0.12
Severe ALD n (%)	14, 40%	19, 61%	0.08
Cirrhosis, n (%)	11, 31%	18, 58%	*p* < 0.05
AH, n (%)	8, 26%	14, 45%	0.05
Severe AH (mDF > 32), n (%)	6, 17%	11, 35%	0.14
Esophageal varices, n (%)	2, 6%	9, 29%	*p* < 0.05
Encephalopathy, n (%)	2, 6%	8, 26%	*p* < 0.05
Jaundice, n (%)	8, 23%	11, 35%	0.26
Ascites, n (%)	11, 31%	13, 42%	0.38

Abbreviations: AH, alcohol-related hepatitis; ALP, alkaline phosphatase; ALT, alanine aminotransferase; AST, aspartate aminotransferase; GGT, gamma-glutamyl transferase INR, International Normalized Ratio; mDF, Maddrey’s modified discriminant function.

**Table 4:** Alcohol consumption parameters and HMGB1 positivity. Where applicable, *p* < 0.05 from U-Mann–Whitney test or Chi-square test were considered significant. IQR—interquartile range. The results are shown as medians (IQR) or counts and percentages. * High-intensity alcohol intake means women/men consuming 8+/10+ drinks in a day.

Variable	HMGB1 Negative *n* = 35	HMGB1 Positive *n* = 31	*p*-Value
Ethanol level during admission, g/L	1.4 (0–3)	0 (0–0)	*p* < 0.01
Positive test for ethanol during admission, *n* (%)	19, 54%	6, 19%	*p* < 0.01
Duration of alcoholic cycle, weeks	9 (3–18)	52 (9–312)	0.08
Duration of sobriety, days	0 (0–1)	4 (0.5–90)	*p* < 0.05
Alcohol units per day, units	20 (8.3–32.7)	15.7 (8–21.7)	0.12
Alcohol abstinence more than one day before admission, *n* (%)	6, 17%	19, 61%	*p* < 0.001
Alcohol withdrawal syndrome, *n* (%)	23, 66%	15, 48%	0.12
High-intensity alcohol intake, *n* (%) *	20, 57%	18, 58%	0.84

## Data Availability

Data are available on demand.

## Abbreviations

The following abbreviations are used in this manuscript:

ABIC	Age–Bilirubin–INR–Creatinine Score
AH	Alcohol-related hepatitis
ALD	Alcohol-Associated Liver Disease
ALI	Alcohol-induced liver injury
ALP	Alkaline Phosphatase
APTT	Activated Partial Thromboplastin Time
AUD	Alcohol Use Disorder
BMI	Body Mass Index
bpm	beats per minute
CLIF-C ACLF	Chronic Liver Failure Consortium—Acute-on-Chronic Liver Failure
CLIF-C AD	Chronic Liver Failure Consortium—Acute Decompensation
CRP	C-Reactive Protein
DAMPs	Damage-Associated Molecular Patterns
DNA	Deoxyribonucleic Acid
DBP	Diastolic Blood Pressure
GGT	Gamma-Glutamyl Transferase
HMGB1	High-Mobility Group Box 1
HR	Heart Rate
INR	International Normalized Ratio
IQR	Interquartile Range
MAFLD	Metabolic Dysfunction-Associated Fatty Liver Disease
MELD	Model for End-Stage Liver Disease
mDF	Maddrey’s modified discriminant function
NAFLD	Non-Alcoholic Fatty Liver Disease
PLT	Platelets
PT	Prothrombin Time
PTMs	Post-translational modifications
RAGE	Receptor for Advanced Glycation End Products
SBP	Systolic Blood Pressure
TLR	Toll-Like Receptor
TNF-α	Tumor Necrosis Factor Alpha
WBC	White Blood Cells
